# Giant cell glioblastoma is a distinctive subtype of glioma characterized by vulnerability to DNA damage

**DOI:** 10.1007/s10014-019-00355-w

**Published:** 2019-10-26

**Authors:** Kaoru Ogawa, Akira Kurose, Akihisa Kamataki, Kenichiro Asano, Kosuke Katayama, Hidekachi Kurotaki

**Affiliations:** 1 Department of Anatomic Pathology, Hirosaki University Graduate School of Medicine, 5 Zaifu, Hirosaki, 036-8562 Japan; 2 Department of Neurosurgery, Hirosaki University Graduate School of Medicine, Hirosaki, Japan; 3 Department of Pathology, Aomori Prefectural Central Hospital, Aomori, Japan

**Keywords:** Giant cell glioblastoma, DNA double-strand breaks, *TERT*, OLIG2, Ki67

## Abstract

Giant cell glioblastoma (GC-GBM) consists of large cells with pleomorphic nuclei. As a contrast to GC-GBM, we defined monotonous small GBM (MS-GBM) as GBM that consists of small cells with monotonous small nuclei, and compared the DNA damage as well as other pathological features. GC-GBM showed minimal invasion (< 2 mm) and focal sarcomatous areas. *TERT*p was wild type in GC-GBM but mutant in MS-GBM. OLIG2 expression was significantly higher in MS-GBM (*P *< 0.01) (77% in MS-GBM and 7% in GC-GBM). GC-GBM showed significantly higher DNA double-strand breaks (DSBs) compared with MS-GBM (*P *< 0.01) (76% in GC-GBM and 15% in MS-GBM). Nearly, all large cells in GC-GBM underwent DSBs. Thus, significant DSBs in GC-GBM might be induced by an innate lesser stemness characteristic and be followed by mitotic slippage, resulting in polyploidization and the large pleomorphic nuclei. We conclude that GC-GBM is a distinctive subtype of glioma characterized by its vulnerability to DNA damage and that wild-type *TERT*p and lower OLIG2 function might induce this feature. Notably, even large pleomorphic nuclei with severe DSBs demonstrated Ki67 positivity, which alerts pathologists to the interpretation of Ki67 positivity, because cells with large nuclei undergoing severe DSBs cannot be recognized as proliferating cells that contribute to tumor aggressiveness.

## Introduction

Glioblastoma (GBM) is the most frequent malignant brain tumor and its prognosis is poor regardless of its subtype. While GBM commonly shows dense proliferation of highly atypical and pleomorphic cells, necrosis, and microvascular proliferation, its morphology greatly varies from case to case. GBM is also characterized by rapid growth and diffuse infiltration into surrounding brain tissues. GBM usually consists of pleomorphic cells, but even classic GBM occasionally shows diffuse and dense proliferation of small cells with monotonous small and hyperchromatic nuclei.

Giant cell GBM (GC-GBM), which is listed among the GBM subtypes defined by the latest World Health Organization (WHO) classification [1], is a peculiar subtype of GBM made up of large cells that exhibit marked nuclear pleomorphism. The nuclei are large and demonstrate severe atypia, often resulting in bizarre nuclei. Multinucleated giant cells are commonly seen. In GC-GBM, *TP53* mutations are often observed, whereas mutations of *IDH* and *TERT* promoter (*TERT*p) are uncommon [2, 3]. Lack of *BRAF*^V600E^ mutation is a discriminating feature of GC-GBM from pleomorphic xanthoastrocytoma [4–6]. Although GC-GBM shows better prognosis compared with other classic GBM subtypes [7], the molecular biology of GC-GBM has not yet been sufficiently elucidated.

In the present study, we investigated GC-GBM, focusing on elucidation of the mechanism that induces the characteristic nuclei of GC-GBM as well as morphological features. We speculated that GC-GBM has less stemness and easily induces pleomorphic nuclei. On the other hand, GBM that demonstrates high cellularity consisting of small cells with small monotonous hyperchromatic nuclei has sufficient stemness characteristics for the maintenance of these nuclei as well as aggressive proliferation.

To study this hypothesis, we defined GBM exhibiting dense proliferation of small cells with small monotonous hyperchromatic nuclei as monotonous small GBM (MS-GBM) and compared GC-GBM with MS-GBM. First, we observed a degree of DNA double-strand breaks (DSBs) in the two groups using anti-γH2AX antibody. Recent research has shown that phosphorylation of histone H2AX, one of the variants of the nucleosome core histone H2A, can be a reliable marker of DNA DSBs. DNA DSBs induce phosphorylation of histone H2AX on Ser-139; phosphorylated H2AX is defined as γH2AX [8]. This phosphorylation event takes place on H2AX molecules on both sides of DSBs along a megabase length of DNA [8]. γH2AX can be detected immunocytochemically and the degree of DNA DSBs correlates with the positivity [9–11]. Using γH2AX, we conducted a prior study: induction of severe DNA DSBs in cultured GBM cells caused mitotic slippage and nuclear enlargement without undergoing apoptosis, resulting in senescence [12]. Therefore, we predicted that the pleomorphic large nuclei noted in GC-GBMs might be caused by severe DNA DSBs, and explored both the genetic and phenotypic features of GC-GBM in comparison with MS-GBM in this study.

## Materials and methods

Cases were selected among patients who underwent brain surgery between 2011 and 2018 at our university and a related hospital. According to the WHO classification [1], five cases (Cases 1–5) of GC-GBM were selected. We defined monotonous small GBM (MS-GBM) as GBM consisting of dense proliferation of small cells with monotonous small and hyperchromatic nuclei, which morphologically contrasted to GC-GBM. For MS-GBM, five cases (Cases 6–10) were selected (Table 1).

**Table 1:**
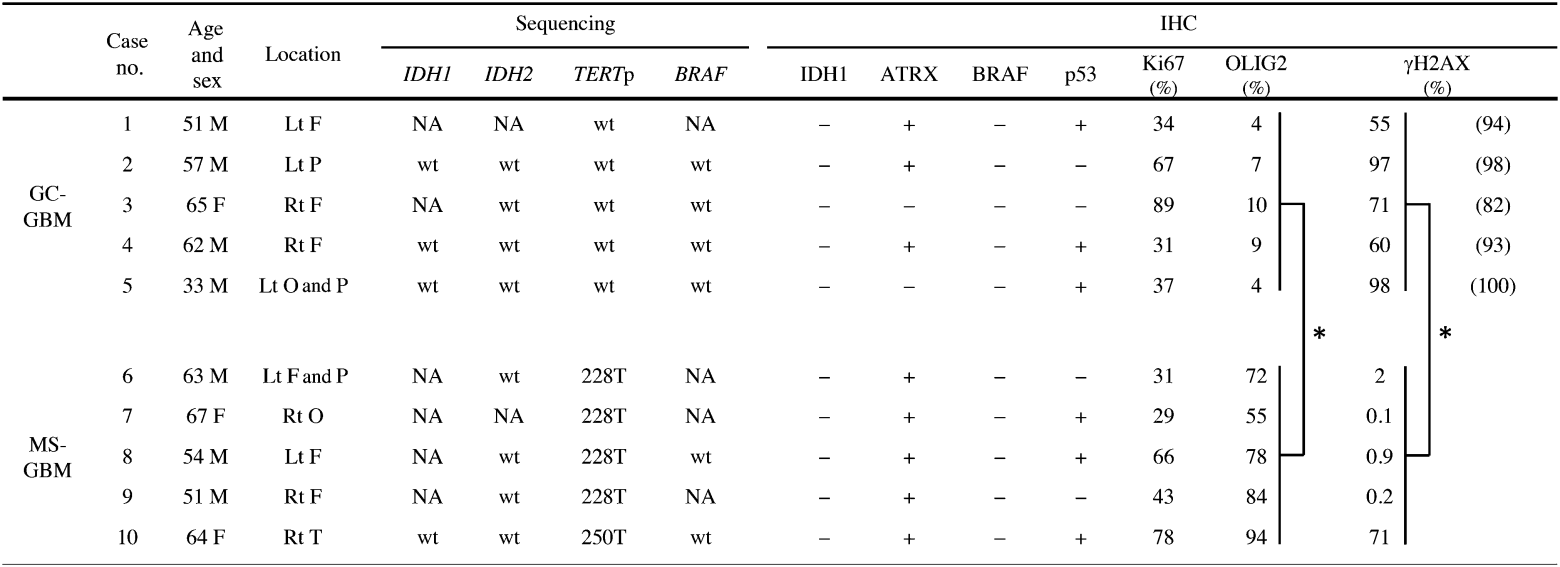
Summary of clinical information, DNA sequencing, and IHC study

Numbers within () in the γH2AX lane show percentage of γH2AX-positive cells among cells of which nuclei 2.5 times larger than those of small cells

*F* frontal, *P* parietal, *O* occipital, *T* temporal, *NA* not available, *wt* wild type

*P < 0.01

Excised brain tissue specimens were fixed in 10% buffered formalin within 48 h and routinely processed into formalin-fixed paraffin-embedded (FFPE) blocks before H&E staining. Immunohistochemistry (IHC) using a Leica BOND-III (Leica, Newcastle Upon Tyne, UK) after antigen retrieval on 4-µm-thick FFPE sections was conducted using antibodies against IDH1 (H09, 1:200; Dianova, Hamburg, Germany), ATRX (polyclonal, 1:1000; ATRAS Antibodies, Stockholm, Sweden), BRAF V600E (VE1, 1:50; Spring Bioscience, Pleasanton, CA), GFAP (6F2, 1:400; DAKO, Carpinteria, CA), OLIG2 (polyclonal, 1:300; IBL, Takasaki, Japan), p53 (DO-7, prediluted; Leica), Ki67 (MIB1, 1:200; DAKO), γH2AX (JBW301, 1:400; Merck Millipore, Temecula, CA), CD133 (W6B3C1, 1:10; Miltenyi Biotec, Bergisch Gladbach, Germany), CD44 (DF 1485, 1:30; Abnova, Taipei, Taiwan), Nestin (10C2, 1:200; Merck Millipore), and podocalyxin-like protein (PODXL) (EPR9518, 1:1000; Abcam plc, Cambridge, UK). IHC specimens were visualized using a BOND Polymer Refine Detection kit (Leica).

The proportion of cells positive for Ki67, OLIG2, and γH2AX were calculated after counting more than 1000 consecutive cells in a hot spot (Ki67) or a representative area (OLIG2 and γH2AX) of each case and moderate or strong nuclear staining was estimated to be positive. Moreover, the proportion of positive cells for γH2AX limited to large nuclei that were 2.5 times larger than small nuclei was also calculated. GFAP was evaluated as the total positive area per total tumor area. Positive estimation of p53 was elicited when more than 10% of tumor cells showed strong immunoreactivity [13]. *P* values were calculated using a two-sided paired *t* test.

For double staining of Ki67 and γH2AX on GC-GBM sections, primary antibodies against γH2AX (JBW301, 1:200; Merck Milipore) and Ki67 (rabbit monoclonal EPR3610, 1:250; Abcam) followed by alkaline phosphatase-conjugated goat anti-rabbit IgG (polyclonal, 1:200; Nichirei Biosciences Inc., Tokyo, Japan) and peroxidase-conjugated goat anti-mouse IgG (polyclonal, 1:200; Nichirei Biosciences Inc.) secondary antibodies were used and observed microscopically.

For sequencing, genomic DNA was isolated from FFPE or fresh samples using a QIAamp DNA FFPE Tissue Kit (Qiagen, Valencia, CA) or a QIAamp Fast DNA Tissue Kit (Qiagen). For *IDH*1/2, *TERT*p, and *BRAF*^V600^, amplification was performed with the primers listed below [14] and KOD-Plus ver.2 (TOYOBO,) using a Veriti 96-Well Thermal Cycler (Applied Biosystems, Inc., Waltham, MA).

**Table:** 

IDH1	Forward	5′-caaatgtgccactatcactcc-3′
	Reverse	5′-gttggaaatttctgggccatg-3′
IDH2	Forward(+M13M3)	5′-tgtaaaacgacggccagtggttgaaagatggcggctg-3′
	Reverse	5′-tgtggccttgtactgcagag-3′
TERT (FFPE)	Forward	5′-tcctgccccttcaccttccag-3′
	Reverse	5′-acgcagcgctgcctgaaactc-3′
TERT (Fresh)	Forward(+M13M3)	5′-tgtaaaacgacggccagtggccgattcgacctctct-3′
	Reverse	5′-agcacctcgcggtagtgg-3′
BRAF	Forward	5′-atctcacctcatcctaacac-3′
	Reverse(+M13RV)	5′-caggaaacagctatgacatggatccagacaactgttc-3′

The amplicon was gel-purified using a QIA quick PCR Purification Kit (QIAGEN). Together with the primers listed below [14, 15] and a Big Dye Terminator v1.1 Cycle Sequencing Kit (Applied Biosystems), target DNA was amplified using a Veriti 96-Well Thermal Cycler.

**Table:** 

IDH	Forward	5′-ctcctgatgagaagagggttg-3′
	Reverse	5′-cacattattgccaacatgac-3′
IDH2	Forward(+M13 primer M3)	5′-tgtaaaacgacggccagt-3′
	Reverse	5′-tgtggccttgtactgcagag-3′
TERT (FFPE)	Forward	5′-tcctgccccttcaccttccag-3′
	Reverse	5′-acgcagcgctgcctgaaactc-3′
TERT (Fresh)	Forward(+M13 primer M3)	5′-tgtaaaacgacggccagt-3′
	Reverse	5′-agcacctcgcggtagtgg-3′
BRAF	Forward	5′-atctcacctcatcctaacac-3′
	Reverse(+M13 primer RV)	5′-caggaaacagctatgac-3′

Sequencing was performed using an ABI PRISM 310 genetic analyzer (Applied Biosystems) or a 3500 Genetic analyzer (Applied Biosystems).

### Ethics statement

This study was approved by the institutional ethics committees at Hirosaki University Hospital (No. 2018-128) and Aomori Prefectural Central Hospital (No. 166-7), and it complied with all provisions of the Declaration of Helsinki.

## Results

**Fig. 1:**
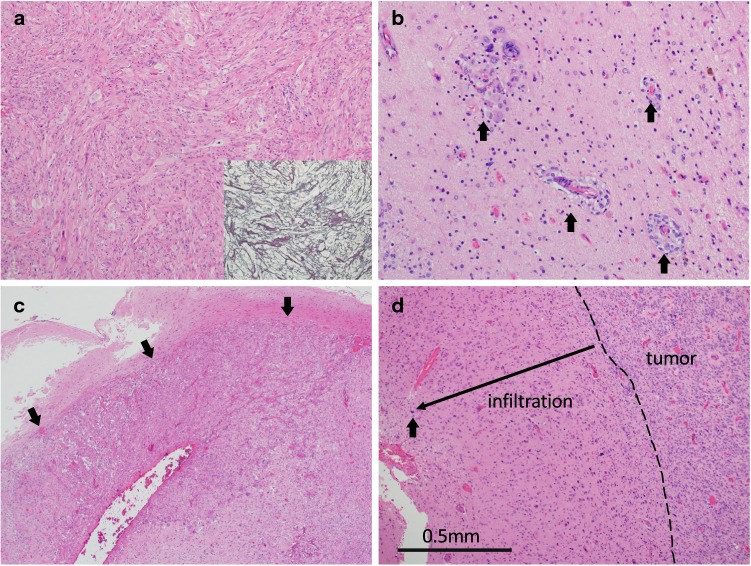
Histological findings of GC-GBM. **a** Sarcomatous area with reticulin stain (insert) in Case 1. **b** Extension through the Virchow–Robin spaces (arrows) around the main tumor in Case 5. **c, d** Clear tumor border (arrows) (**c**) and minimum infiltration (**d**) in Case 4. Bold arrow shows the most distant tumor cell from the main tumor (**d**)

GC-GBM mainly consisted of large cells with single or multiple large pleomorphic nuclei intermingled with bizarre ones. Lobulated nuclei were frequently seen. Small cells with small oval nuclei were also observed and their ratio varied depending on area. MS-GBM showed a dense proliferation of small cells with small hyperchromatic nuclei. Cases 9 and 10 of MS-GBM were diagnosed as small-cell GBM according to the WHO classification [1] because of bland nuclei and chicken wire-like microvasculature, and the others (Cases 6, 7, and 8) were classic GBM. Table 2 details the pathological findings of GC-GBM. Geographic necrosis was observed in all GC-GBM cases and four cases (Cases 1, 2, 4, and 5) also showed palisading necrosis, while geographic necrosis was dominant. Microvascular proliferation was noted in four of five cases (Cases 2, 3, 4, and 5) and glomeruloid structures were concomitant in two cases (Cases 2 and 3). Sarcomatous areas showing proliferation of spindle-shaped cells (Fig. 1a) were evident at least in part of all cases of GC-GBM and networks of reticulin fibers were concomitant in two cases (Cases 1 and 5) (Fig. 1a). Specimens of all cases of GC-GBM contained non-neoplastic cerebral tissues adjacent to the tumor and borders of the tumor were clear or minimally invasive (less than 2 mm) in all the cases of GC-GBM (Fig. 1c, d). However, extension of GBM cells through the Virchow–Robin spaces (Fig. 1b) adjacent to the main tumor was seen in one case (Case 5) of GC-GBM. Positive areas for GFAP in GC-GBM varied from 10 to 90% (data not shown).

**Table 2:** Pathological findings of GC-GBM

Case no.	Necrosis	MVP	Sarcomatous area	Reticulin fiber	Clear border or minimal invasion
*GC*-*GBM*					
1	GN > PN	−	+	+	+
2	GN > PN	+ (GS+)	+	−	+
3	GN	+ (GS+)	+	−	+
4	GN > PN	+	+	−	+
5	GN > PN	+	+	+	+

*GN* geographic necrosis, *PN* palisading necrosis, *MVP* microvascular proliferation, *GS* glomeruloid structure

In MS-GBM, palisading necrosis was observed in four of five cases (Cases 6, 8, 9, and 10) and Case 10 showed widespread of palisading necrosis throughout the tumor tissue.

DNA sequencing and IHC results are summarized in Table 1. In GC-GBM, three cases (Cases 2, 4, and 5) had wild-type *IDH* by sequencing. FFPE materials of two cases (Cases 1 and 3) that were not suitable for sequencing showed negative IDH1 immunohistochemical staining.

Codons 228 and 250 of the *TERT*p gene were wild type in all the cases of GC-GBM and mutant in all the cases of MS-GBM. *BRAF*^V600^ was wild type in four of five cases (Cases 2, 3, 4, and 5) of GC-GBM and Case 1 was negative for BRAF^V600E^ immunohistochemically.

**Fig. 2:**
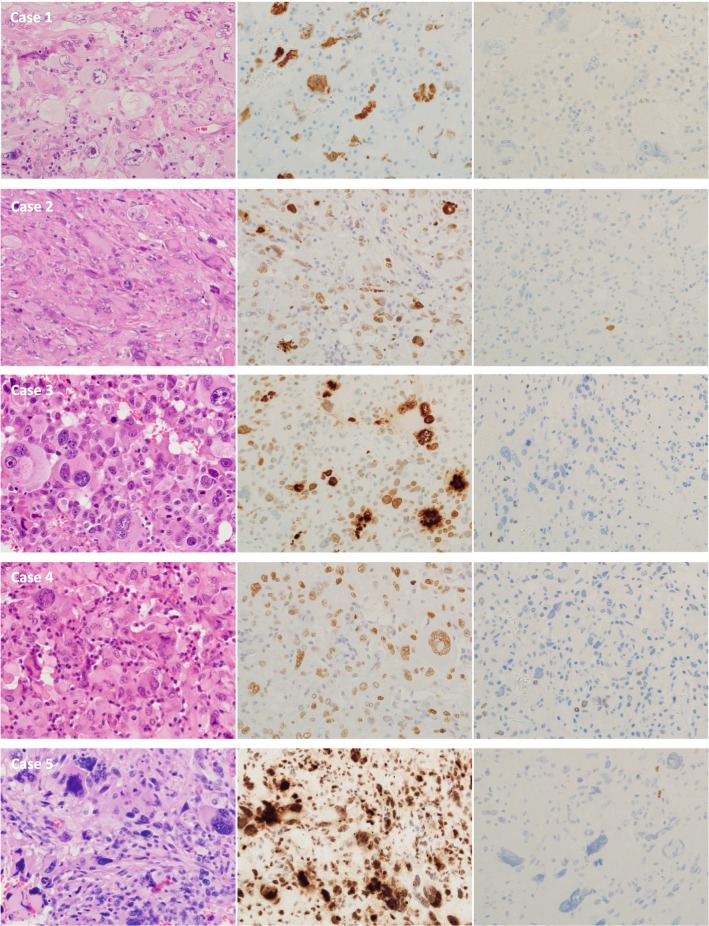
Cases 1–5 of GC-GBM with IHC for γH2AX (middle lane) and OLIG2 (right lane)

**Fig. 3:**
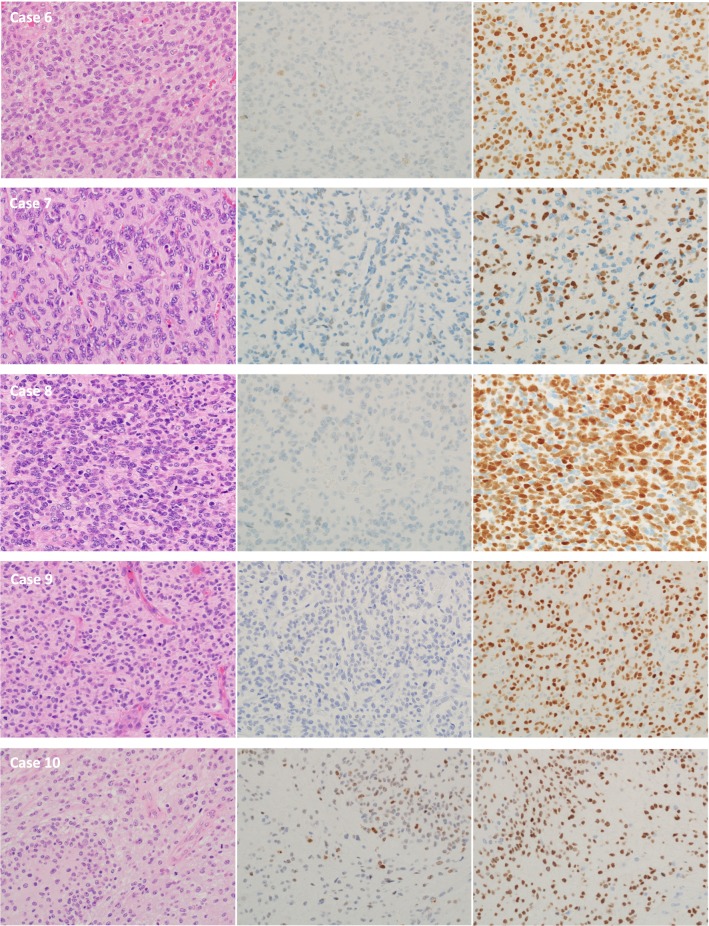
Cases 6–10 of MS-GBM with IHC for γH2AX (middle lane) and OLIG2 (right lane)

DNA DSBs detected by IHC for γH2AX were markedly seen mainly in the large nuclei of all cases of GC-GBM (Fig. 2). Average rates of γH2AX-positive cells were 76% (range 55–98%) in GC-GBM and 15% (range 0.1–71%) in MS-GBM (Fig. 3, Table 1); the positive rate was significantly higher in GC-GBM (*P *< 0.01). The nuclei 2.5 times larger than the small nuclei of GC-GBM demonstrated marked positivity for γH2AX and an average rate of 93% (range 82–100%).

In the four cases (Cases 6, 7, 8, and 9) of MS-GBM, positivity for γH2AX was not more than 2%. Only Case 10, which demonstrated widespread palisading necrosis throughout the tumor tissues, showed 71% positivity. Hot spots for γH2AX of Case 10 were contained in the palisading areas and the positive rate decreased to 10–20% as it separated away from the palisading areas. In all cases of MS-GBM, nuclear positivity for γH2AX was faint to moderate.

Average positivity rates for Ki67 were similar between GC-GBM (52%; range 31–89%) and MS-GBMs (49%; range 29–78%). In all cases of GC-GBM, Ki67 was demonstrated even in large, bizarre, or lobulated nuclei (Fig. 5a). Double staining for Ki67 and γH2AX demonstrated that a certain number of Ki67-positive cells also showed γH2AX (Fig. 5b).

Average positivity rates for OLIG2 were 7% (range 4–10%) in GC-GBM and 77% (range 55–94%) in MS-GBM; the positivity rate was significantly lower in GC-GBM compared with MS-GBM (*P *< 0.01) (Table 1).

**Fig. 4:**
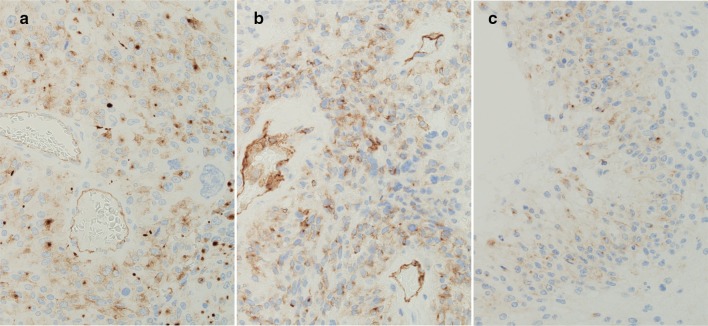
IHC for PODXL in Cases 3 (**a**) and 5 (**b**) in GC-GBM, and Case 10 (**c**) of MS-GBM

Immunohistochemically, CD133, CD44, and nestin expression was positive in tumor cells regardless of cell size in GC-GBM, and there was no site-dependent propensity in MS-GBM. PODXL was focally positive mainly in small cells around the small vessels in Cases 3 and 5 and the palisading areas in Case 10 (Fig. 4).

## Discussion

GC-GBM demonstrated a relatively clear tumor border that was linear (Fig. 1c) or with minimum invasion of less than 2 mm (Fig. 1d), even if infiltration into surrounding brain tissues was observed. Focal sarcomatous areas, which have not been emphasized thus far, were seen in all of the GC-GBM cases. Minimum invasion and focal sarcomatous areas can be considered to be additional features for GC-GBM.

The gene status of GC-GBM using FFPE showed wild-type *IDH* in three of five cases. For Case 3, FFPE samples could not provide the gene status of *IDH1* and were negative for IDH1 by IHC. Thus, Case 3 was estimated as wild type for *IDH,* because the patient was over 55 years old. Therefore, at least four of five cases of GC-GBM were wild type for *IDH.* The results of sequencing and IHC indicate that GC-GBM is characterized by wild-type *IDH*, *TERT*p, and *BRAF* V600.

Anti-γH2AX antibody is an excellent marker for DNA DSBs and degree of DNA DSBs correlates with immunocytochemical detection [8, 16]. IHC using anti-γH2AX antibody demonstrated a marked number of cells with severe DNA DSBs in GC-GBM.

In MS-GBM, four cases (Cases 6, 7, 8, and 9) contained few cells with DNA DSBs (0.1–2%). Case 10 demonstrated a marked number of cells in palisading areas with DNA DSBs, and the cells with DNA DSBs decreased in number away from the palisading areas. Since in the cases of MS-GBM other than Case 10, an increase in the number of cells with DNA DSBs in palisading areas was not evident, Case 10 might lack a cellular function against DNA damage induced by ischemia, which is usually present in MS-GBM.

In GC-GBM, significantly higher number of cells showed DNA DSBs compared with MS-GBM (*P *< 0.01). Moreover, nearly all large nuclei in GC-GBM showed severe DNA DSBs. On the other hand, MS-GBM (other than Case 10) seldom showed DNA DSBs and the degree of DNA DSBs was faint to moderate.

We examined DNA DSBs with IHC using anti-γH2AX antibody in every GBM case (data not shown) and found that γH2AX positivity in classical GBM cases varied from case to case (nearly 0% to approximately 80%). However, the degree of positivity was mainly faint to moderate. Although pleomorphic large cells in classical GBM usually showed positivity for γH2AX, the number was far from those of GC-GBM. These findings suggest that GC-GBM and MS-GBM are sharply contrasting and distinct groups of GBM in DNA DSBs.

Given these findings, it is plausible that MS-GBM is usually equipped with protective functions against DNA damage, while its vulnerability to DNA damage, which can cause large pleomorphic nuclei with significant DNA DSBs, is possibly the most characteristic features of GC-GBM compared with the other types of GBM.

To compere the protective function against DNA damage in GC-GBM and MS-GBM, we studied the expression of stem cell markers, because some have been shown to function against DNA damage. To detect stem cells in GBM, many types of markers have been reported [17, 18]. Among markers stained in the present study, PODXL [19] showed a propensity for small cells around vessels (Cases 3 and 5) and in palisading areas (Case 10) (Fig. 4). Although we could not sufficiently show that the small cells in GC-GBM included stem cells according to their markers, the concept that large cells with large pleomorphic nuclei can hardly be considered to be stem cells indicate that stem cells exist among small cells other than cells with nuclear pleomorphism in GC-GBM. Therefore, it is suggested that since GC-GBM has an innate feature of insufficient protection against DNA damage, proliferating cells derived from stem cells can easily suffer from DNA DSBs induced by cell metabolism and, thus, lose stemness. On the other hand, MS-GBM might be equipped with enough protective function against DNA damage to maintain stemness and hence exhibit a similar morphology to stem cells.

Successful mitosis can be achieved after proper chromosomal segregation and cytokinesis at the mitotic phase. It is strongly suggested that severe DNA DSBs result in failure of chromosome segregation and the subsequent cytokinesis in GC-GBM. Consequently, large cells with large pleomorphic nuclei or multinucleated giant cells characteristic of GC-GBM might emerge.

**Fig. 5:**
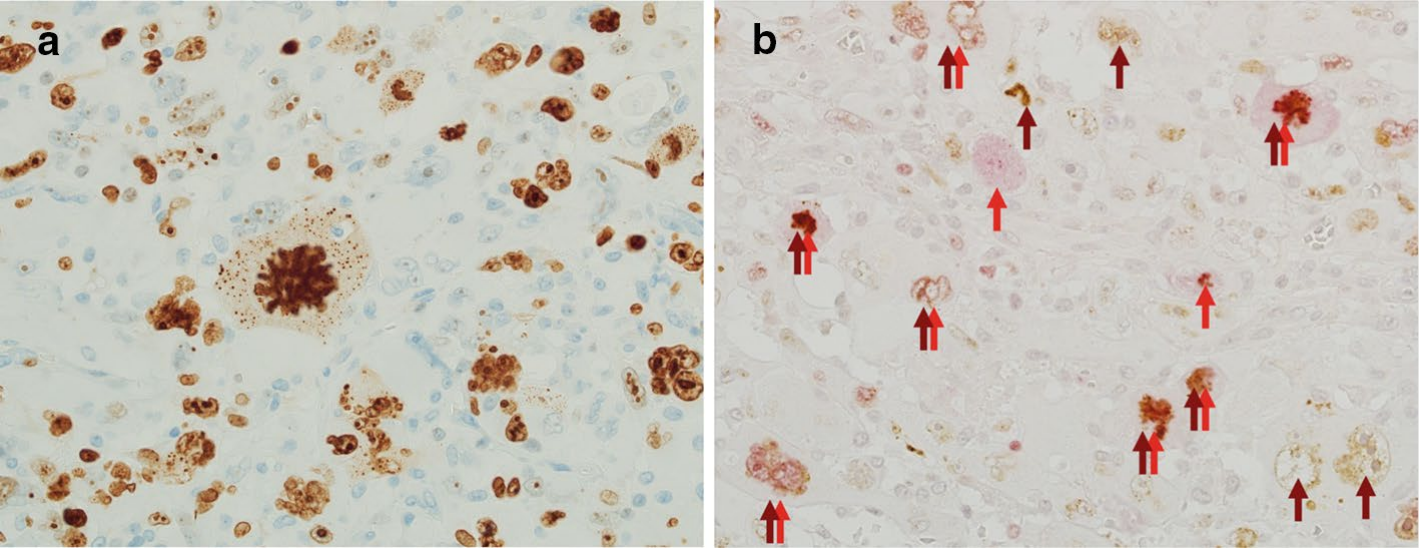
IHC for Ki67 and γH2AX in Case 1. **a** Large, bizarre, or lobulated nuclei as well as the nuclei of multinucleated cells were positive for Ki67. **b** Double staining for Ki67 (red arrows) and γH2AX (brown arrows). Of nine nuclei positive for Ki67, seven were also positive for γH2AX

It was noted that a certain number of large cells with large pleomorphic nuclei positive for Ki67 underwent severe DNA DSBs (Fig. 5). Such large cells with pleomorphic nuclei cannot be considered as proliferating cells that contribute to tumor aggressiveness. In the present study, although positivity for Ki67 was similar between GC- and MS-GBM cases, cycling cells reflecting tumor growth should be at much lower levels in GC-GBM. These observations alert pathologists to estimate the percentage of Ki67-positive cells; Ki67 positivity does not necessarily correlate with tumor aggressiveness.

We previously showed that a cell line of GBM underwent severe DNA DSBs after temozolomide treatment and then bypassed cytokinesis, resulting in tetraploidization and senescence [12]. Like this phenomenon, it is suggested that tumor cells derived from stem cells in GC-GBM undergo mitotic spillage [20] and tetra- or polyploidization, eventually resulting in possible senescence. This can explain the better prognosis of GC-GBM compared with classic GBM [7].

It is well known that mutations of *TERT*p lead to increased telomerase expression and immortalization [21]. Moreover, there have been reports that *TERT*p mutations facilitate the repair of DNA DSBs [22] and enable escape from senescence or apoptosis [23]. The findings that all cases of GC-GBM and MS-GBM had wild-type and mutant *TERT*p, respectively, might indicate that *TERT*p mutations are correlated with a protective function against DNA damage as well as maintenance of stemness.

OLIG2, which exhibited significantly different expression between GC-GBM and MS-GBM, is commonly expressed in glioma [24] and has also been identified as a transcription factor that reprograms differentiated GBM cells into stem-like cells [25]. Higher expression of OLIG2 in MS-GBM might be associated with a stemness characteristic of MS-GBM compared with GC-GBM.

We showed that most large cells characterized as GC-GBM underwent severe DNA damage. Conversely, MS-GBM has a characteristic that protects against DNA damage through an unknown mechanism absent in GC-GBM that is possibly correlated with *TERT*p mutations and OLIG2 function. Thus, MS-GBM might maintain stemness and the small-cell morphology that resembles stem cells.

Although cases analyzed in this study were limited and further research is required, it can be suggested that due to insufficient protection against DNA damage, the majority of tumor cells derived from stem cells of GC-GBM easily suffer from DNA DSBs and undergo mitotic slippage, thus resulting in the ‘giant cell’ features of GC-GBM.
